# Farm production, market access and dietary diversity in Malawi

**DOI:** 10.1017/S1368980016002135

**Published:** 2016-09-09

**Authors:** Stefan Koppmair, Menale Kassie, Matin Qaim

**Affiliations:** 1 Department of Agricultural Economics and Rural Development, Georg-August-University of Goettingen, Platz der Göttinger Sieben 5, 37073 Goettingen, Germany; 2 International Centre of Insect Physiology and Ecology (ICIPE), Nairobi, Kenya

**Keywords:** Nutrition-sensitive agriculture, Dietary diversity, Agricultural technology, Smallholder farmers, Malawi

## Abstract

**Objective:**

The association between farm production diversity and dietary diversity in rural smallholder households was recently analysed. Most existing studies build on household-level dietary diversity indicators calculated from 7d food consumption recalls. Herein, this association is revisited with individual-level 24 h recall data. The robustness of the results is tested by comparing household- and individual-level estimates. The role of other factors that may influence dietary diversity, such as market access and agricultural technology, is also analysed.

**Design:**

A survey of smallholder farm households was carried out in Malawi in 2014. Dietary diversity scores are calculated from 24 h recall data. Production diversity scores are calculated from farm production data covering a period of 12 months. Individual- and household-level regression models are developed and estimated.

**Setting:**

Data were collected in sixteen districts of central and southern Malawi.

**Subjects:**

Smallholder farm households (*n* 408), young children (*n* 519) and mothers (*n* 408).

**Results:**

Farm production diversity is positively associated with dietary diversity. However, the estimated effects are small. Access to markets for buying food and selling farm produce and use of chemical fertilizers are shown to be more important for dietary diversity than diverse farm production. Results with household- and individual-level dietary data are very similar.

**Conclusions:**

Further increasing production diversity may not be the most effective strategy to improve diets in smallholder farm households. Improving access to markets, productivity-enhancing inputs and technologies seems to be more promising.

Despite substantial improvements in food security over the last few decades, undernutrition remains a global burden. Approximately 800 million people are chronically hungry, most of them living in developing countries^(^ ^1^ ^)^. An estimated two billion people suffer from deficiencies in particular micronutrients, such as iron, zinc or vitamin A^(^ ^2^ ^)^. Nutritional deficiencies harm physical and mental human development, increase the susceptibility to infectious diseases and contribute to premature deaths. Women and children pay the heaviest toll. Forty-five per cent of all deaths of children under 5 years of age are linked to undernutrition^(^ ^2^ ^)^. Overall, undernutrition is the cause of 3·1 million child deaths annually^(^ ^3^ ^)^. Childhood undernutrition also decreases adult productivity and entails substantial economic losses in many developing countries^(^ ^2^ ^)^.

Nutrition is closely linked to agriculture, not only because agriculture is the sector that produces food, but also because many of the undernourished people worldwide are smallholder farmers^(^ ^4^ ^,^ ^5^ ^)^. For a long time, the main agricultural policy response to undernutrition was to strengthen staple food production through price incentives and promoting improved farm technologies. The focus was primarily on a narrow range of cereal crops, especially wheat, rice and maize^(^ ^6^ ^)^. While this strategy has clearly helped to reduce hunger, it has also contributed to lower levels of crop species diversity^(^ ^7^ ^)^. More homogeneous global food supplies may have decreased dietary diversity^(^ ^8^ ^,^ ^9^ ^)^. And low levels of dietary diversity are associated with higher rates of micronutrient deficiencies, child stunting, child deaths and other negative health consequences^(^ ^10^ ^–^ ^13^ ^)^.

More diversified agricultural and food systems may help to improve dietary quality and nutrition^(^ ^6^ ^,^ ^14^ ^–^ ^16^ ^)^, but empirical evidence on the effects of diversification strategies on dietary improvement in smallholder households is scarce^(^ ^17^ ^)^. Appropriate levels of diversification are a question of scale. Food systems diversity does not necessarily imply that every single farm has to be extremely diverse. On the one hand, diverse farm production may promote diverse food consumption in the farm household. This is especially true in sub-Saharan Africa, where smallholder farms are often subsistence-oriented^(^ ^18^ ^)^. On the other hand, typical farms in Africa are already quite diverse. Further diversification might prevent gains from specialization on the farm and could thus result in income losses, with potential negative nutritional effects^(^ ^19^ ^)^. In spite of their subsistence orientation, smallholder farm households are engaged in market transactions. A substantial share of the food consumed in farm households is purchased from the market^(^ ^20^ ^–^ ^23^ ^)^. In addition, foods obtained through hunting, fishing or collection from the natural environment can play a significant role for dietary diversity in rural areas^(^ ^24^ ^–^ ^26^ ^)^.

Recent studies have empirically analysed the link between farm species diversity and dietary diversity in a number of developing countries^(^ ^19^ ^,^ ^27^ ^–^ ^29^ ^)^. While the exact estimates differ, a significant but relatively small positive relationship was generally found. Yet, the same studies also pointed out that market access may be a more important factor influencing dietary diversity in smallholder farm households. These results have stirred an interesting debate^(^ ^30^ ^–^ ^32^ ^)^. In particular, questions about the indicators used to measure production and consumption diversity were raised.

We contribute to this emerging literature on the link between farm production diversity and dietary diversity by using alternative indicators and comparing results. Previous studies used food consumption data to construct dietary diversity scores at the household level^(^ ^19^ ^,^ ^27^ ^–^ ^29^ ^)^. The use of household-level consumption data is convenient, because such data are often available from nationally representative living standard measurement surveys. Living standard surveys often include a 7d or 30d consumption recall that can be used to construct dietary indicators. However, from a nutritional perspective, shorter recall periods are generally preferred^(^ ^33^ ^)^. Moreover, household-level data do not account for issues of intra-household distribution and therefore cannot be used for statements concerning particular population groups, such as children. Herein, we use data from a 24 h dietary recall carried out at household and individual levels to analyse and compare the relationship between farm production diversity and dietary diversity. Furthermore, beyond measuring farm diversity in terms of a simple count of the species produced, we construct production diversity scores that better account for nutritional functions^(^ ^27^ ^,^ ^30^ ^)^. Finally, in comparison to previous studies we use a larger set of variables to estimate the role of market access and agricultural technology.

For the empirical analysis, we use data from a recent survey of farm households in Malawi, covering household- and individual-level information. Malawi is an interesting study country for several reasons. First, Malawi is poor with high rates of undernutrition^(^ ^34^ ^)^. Second, farm households in Malawi are primarily subsistence-oriented. Third, several previous studies on the link between farm production and dietary diversity used household-level data from Malawi’s Living Standards Measurement Survey^(^ ^19^ ^,^ ^28^ ^,^ ^29^ ^)^. Focusing on the same setting with individual-level data and alternative indicators has advantages in terms of comparability.

## Materials and methods

### Data

Data for the present study come from a farm household survey that we conducted in cooperation with the International Maize and Wheat Improvement Center (CIMMYT) and the Malawian Department of Agricultural Research Services in January and February 2014. The survey covered sixteen districts and 165 villages throughout the country’s central and southern regions. Household selection was based on a multistage proportionate random sampling procedure. Interviews captured a wide array of information, including details on household demographics, household socio-economic status, agricultural production and marketing and consumption of food and non-food products. A special section with a 24 h food consumption recall captured dietary patterns of all household members combined, as well as individually for children below the age of 5 years and their mothers.

The survey team included experienced local enumerators who had been selected and trained intensively. Sensitive sections, such as the 24 h consumption recall, were especially emphasized during the training and the pre-testing of the questionnaire. Enumerators were carefully trained to not only focus on the main meals consumed in the household, but to also elicit details on snacks and minor dietary components. Overall, 1482 farm households were surveyed. Out of the sampled households, only 408 had children below 5 years of age. We want to compare dietary diversity at household level and individual level for children and mothers, which is most meaningful when focusing on the same households. Hence, the current analysis builds on the 408 households with small children and their mothers. The sample is representative of farm households with small children in central and southern Malawi.

### Analytical approach

To analyse the relationship between farm production diversity and dietary diversity, we use the following regression model:
1

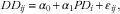

where *DD*_*i*_ is dietary diversity and *PD*_*i*_ is production diversity in farm household *i*. *ε*_*i*_ is a random error term, and *α*_0_ and *α*_1_ are coefficients to be estimated. We are particularly interested in the estimate for *α*_1_. We estimate different versions of this model, changing the measures of *DD* and *PD*, as is further explained below. In one set of models, *DD* is measured at the household level. In alternative specifications, *DD* is measured for individual *j* living in household *i*. In particular, we consider children below 5 years of age and their mothers.

The model in equation (1) includes only production diversity as explanatory variable. Yet, there may also be other factors that could influence dietary diversity, such as market access and other socio-economic and demographic characteristics. To better understand the role of such other factors, we extend the regression model as follows:
2

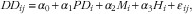

where *M*_*i*_ is a vector of variables capturing market access and *H*_*i*_ is a vector of other socio-economic and demographic variables, including farm size, household size, off-farm income, as well as age, education and gender of the household head. We use different indicators to capture market access and market use for agricultural sales and food purchases of household *i*.

To analyse the role of agricultural technology, we further extend this model as follows:
3

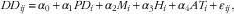

where *AT*_*i*_ represents a vector of dummy variables indicating the use of different types of agricultural technology. Further details of how variables are defined and measured are provided below.

### Measurement of dietary diversity

We measure dietary diversity in terms of dietary diversity scores (DDS), a common indicator that counts the number of food groups consumed over a certain period of time^(^ ^35^ ^–^ ^37^ ^)^. Most previous studies that analysed the relationship between farm production diversity and dietary diversity calculated DDS at the household level, using data from 7 d food consumption recalls^(^ ^19^ ^,^ ^27^ ^–^ ^29^ ^)^. We use 24 h recall data collected for the household as a whole and for children below 5 years of age and their mothers to calculate and compare DDS at household and individual levels. Only very few previous studies have examined the relationship between production and consumption diversity using individual-level DDS^(^ ^22^ ^,^ ^38^ ^,^ ^39^ ^)^.

Our data in Malawi were collected in one single round. Single-round 24 h food consumption recalls have clear limitations, as they are unable to capture day-to-day variation in dietary intakes. While such variation is often relatively low among poor rural households, it cannot be ignored when making nutritional assessments at individual or household level. In such cases, repeated 24 h recalls are required^(^ ^40^ ^)^. In our study, we do not make nutritional assessments for individuals or households but focus on the analysis of population-level associations, for which the general drawbacks of single-round recalls are less problematic.

Another important aspect when analysing dietary patterns is the question of seasonality. In poor rural households, the types and sources of foods consumed can vary significantly over the year, usually following the cycles of crop harvests^(^ ^21^ ^,^ ^20^ ^)^. Our survey was conducted during the months of January and February, before the main maize harvest. This is considered the lean season in Malawi, when the role of purchased foods tends to be more important than during harvest and post-harvest periods^(^ ^41^ ^)^. In addition, fishing and the collection of wild fruits are more important for household diets during the lean season^(^ ^42^ ^–^ ^44^ ^)^. In the 24 h recall, foods from all sources were captured, including any snacks consumed outside the home. However, for result interpretation it needs to be kept in mind that the concrete findings refer to one particular season and cannot be extrapolated to the rest of the year.

For the calculation of DDS, individual food items consumed were clubbed into broader food groups. Many studies consider twelve different food groups, but there is no international consensus on the best number to use^(^ ^45^ ^)^. Sometimes, food groups with low micronutrient densities are excluded to reflect more healthy diets^(^ ^46^ ^)^. Other studies consider a larger number of food groups to analyse dietary patterns in particular situations^(^ ^47^ ^)^. Here, we use the following twelve food groups to calculate DDS at household and individual levels: (i) cereals; (ii) tubers and roots; (iii) vegetables; (iv) fruits; (v) meat and poultry; (vi) eggs; (vii) fish; (viii) pulses, legumes and nuts; (ix) milk and milk products; (x) oils and fats; (xi) sugar and honey; and (xii) miscellaneous, including spices, condiments and beverages^(^ ^46^ ^,^ ^48^ ^)^.

### Measurement of farm production diversity

During the survey, farmers were asked to report details of their farm production for the last 12 months. Almost all farm households in the sample produce maize as the main staple food. In addition, many households also grow other cereals (e.g. sorghum, millet), legumes (e.g. groundnut, beans, cowpeas), roots and tubers (e.g. cassava, sweet potato), several fruits and vegetables (including from household gardens) as well as cash crops such as tobacco and cotton. Small-scale livestock keeping is also common.

Based on the agricultural data, production diversity indicators were calculated. Several recent studies measured production diversity in terms of a simple count of the number of crop species produced on a farm, or a combination of crop and livestock species^(^ ^19^ ^,^ ^29^ ^,^ ^38^ ^)^. However, a simple species count does not necessarily reflect diversity from a dietary point of view. To better account for the dietary perspective, we use a production diversity score defined as the number of food groups produced^(^ ^22^ ^,^ ^27^ ^,^ ^49^ ^)^. To construct the production diversity score, we considered the same twelve food groups that were already explained above. Hence, if a farm produces several species that belong to the same food groups, the production diversity score will be smaller than the simple species count. For comparison, we also show results of models estimated with a simple crop species count as the indicator of farm production diversity.

### Measurement of market access

Markets can play an important role for farm households who act as both sellers and buyers of food and other agricultural commodities. We capture access to two different types of market, namely small local village markets and larger district markets. Local markets are relevant for sales and purchases of smaller quantities, in order to satisfy immediate needs. Local markets also play an important role for fresh fruits, vegetables and dairy products that cannot be stored for longer periods of time. As local markets are not available in every village in Malawi, we construct a dummy variable that takes a value of 1 if such a market exists in the village where a household resides and 0 otherwise. Larger markets are available in every district, usually in the district capital. Farm households use these district markets to sell farm produce and to buy food and non-food items. Reaching district markets usually involves walking a longer distance; hence most households do this only occasionally. We capture access to district markets through distance expressed in walking hours, which is a continuous variable.

These two market access variables describe the market infrastructure conditions a household faces, but there may also be other factors that influence actual market participation. To gain further insights into the role of markets, we define three market participation variables that we use in alternative model specifications. First is the share of maize sold. Maize is the most important staple food in Malawi that almost all farm households produce, often primarily for subsistence purposes. Yet, even subsistence-oriented households often sell some of their maize to buy other goods needed. Second is the share of other food crops sold, such as legumes, fruits, vegetables, etc. Third is the farm area share grown with non-food cash crops, such as tobacco or cotton. Non-food cash crops are entirely sold. In principle, agricultural sales can influence household nutrition in positive and negative ways. Positive effects on dietary diversity could occur when the cash revenues are used to buy food groups that are not produced by the households themselves. Negative effects could occur when less food is produced at home and the cash revenues are not spent on improving nutrition and health.

### Measurement of agricultural technologies

There is a relatively large body of literature that has analysed effects of agricultural technology adoption on farm incomes, but only a few studies have looked more specifically at the link between technology adoption and household food security or nutrition^(^ ^28^ ^,^ ^50^ ^,^ ^51^ ^)^. In Malawi, the government has recently promoted different technologies to sustainably increase agricultural productivity and reduce poverty. On the one hand, this includes modern inputs such as improved crop varieties and chemical fertilizers, which have been promoted through a targeted input subsidy scheme for several years^(^ ^52^ ^)^. On the other hand, there are also efforts to preserve soil fertility through crop diversification and intercropping with legumes^(^ ^53^ ^,^ ^54^ ^)^. To analyse the role of these technologies for dietary diversity, we construct four technology variables: (i) improved maize varieties; (ii) improved legume varieties; (iii) chemical fertilizers; and (iv) maize–legume intercropping (i.e. growing maize and legumes simultaneously on the same plot of land). These variables are defined as dummies taking a value of 1 when the particular technology was adopted and 0 otherwise.

### Regression estimators

The regression models described in equations (1) to (3) above have dietary diversity as the dependent variable. Dietary diversity is a count variable that is not normally distributed. A common approach for count data models is to use a Poisson estimator^(^ ^55^ ^)^. The Poisson estimator assumes equidispersion; that is, the mean and variance of the dependent variable are assumed to be equal. This assumption is often violated and can lead to incorrect standard errors. Overdispersion, where the conditional variance exceeds the conditional mean, is a common phenomenon in many practical applications^(^ ^56^ ^)^. Similarly, underdispersion can also occur in certain situations^(^ ^57^ ^)^. For each model that we estimate, we use an auxiliary regression test^(^ ^56^ ^)^. The test results are reported below. When the null hypothesis of equidispersion (*α*=0) cannot be rejected, we use the standard Poisson estimator. However, for several models the test results suggest underdispersion (*α*<0). In those cases, we use a generalized Poisson estimator that is suitable for modelling count data with underdispersion^(^ ^57^ ^)^.

In Poisson models, the estimated coefficients can be interpreted as semi-elasticities. For more convenient interpretation, instead of the coefficients we report marginal effects for all explanatory variables. In our models, marginal effects describe how the number of food groups consumed changes when the explanatory variables change by one unit. All models are estimated with standard errors corrected for village clusters. Cluster correction controls for possible error term correlation within villages that can result from similarities in environmental or other conditions^(^ ^58^ ^)^.

## Results

### Descriptive statistics

Descriptive statistics for the variables used in the present study are shown in Table 1. The top part of Table 1 shows DDS at the household level, and individually for children and mothers. At the household level, mean DDS is 4·17; that is, the average household has consumed 4·17 food groups during the reference day. Forty per cent of the households have consumed fewer than four food groups; only 10 % have consumed more than six food groups. The most frequently consumed food groups were cereals and vegetables (see Table S1 in the online supplementary material). Fish was consumed by 20 %, meat by 6 %, and eggs and milk or milk products by less than 5 % of the sample households. These patterns point to relatively low levels of dietary diversity among rural households in Malawi during the lean season, when the data were collected.

**Table 1:** Description of variables (408 farm household observations); smallholder farm households, rural central and southern Malawi, 2014

Variable	Description	Mean	sd
Dietary diversity score (DDS)			
Household DDS	Household dietary diversity score	4·17	1·62
Child DDS†	Dietary diversity score of young children (6 months to 5 years)	3·87	1·92
Mother DDS	Dietary diversity score of mothers of young children	4·11	1·67
Farm production diversity			
Production diversity score	Number of different food groups produced on farm	4·88	1·69
Crop species count	Number of different crop species cultivated on farm	5·79	2·89
Market access			
Village market	Village market exists in community (dummy)	0·56	
Time to district market	Distance to the district market in walking hours	1·34	1·13
Market participation			
Share of maize sold	Percentage of total maize production sold	7·38	13·71
Share of other food crops sold	Percentage of other food crop production sold	34·71	32·23
Area share of non-food cash crops	Percentage of farm area cultivated with non-food cash crops	10·97	17·92
Agricultural technologies			
Improved maize varieties	Farm household cultivates improved maize varieties (dummy)	0·81	
Improved legume varieties	Farm household cultivates improved legume varieties (dummy)	0·62	
Chemical fertilizer	Farm household uses chemical fertilizer (dummy)	0·92	
Maize–legume intercropping	Farm household practises maize–legume intercropping (dummy)	0·51	
Other socio-economic and demographic factors			
Livestock	Number of animals kept in tropical livestock units (TLU)	0·88	1·50
Off-farm income	Cash income from off-farm activities (thousand Malawi Kwacha)	91·34	157·16
Farm size	Total area owned in acres	2·89	1·99
Household size	Total number of household members	6·23	2·02
Age of head	Age of the household head in years	40·81	11·91
Male head	Household head is male (dummy)	0·86	
Education of head	Education of the household head in years	5·39	3·42

† The total number of children (below 5 years of age) in the 408 households is 519.

Individual-level DDS are somewhat lower than those measured at the household level. This is expected because at household level all household members’ consumption is covered, including children above the age of 5 years, adolescents, male adults, etc. However, the differences between household- and individual-level DDS are relatively small, and the different measures are strongly correlated. The correlation coefficient between child and household DDS and between mother and household DDS is 0·78 and 0·90, respectively. Within the group of children, we examined whether age and gender have a systematic influence on DDS, but found no significant effects.

The lower part of Table 1 shows the variables that we use as covariates in the different specifications of the regression models. The average farm produces 4·88 different food groups and 5·79 different crop species. In terms of market access, 56 % of the sample households live in villages that have a local market. The average walking distance to the larger district market is 1·34 h. Less than 8 % of the maize produced is sold in the market, underlining that the sample farms are fairly subsistence-oriented. On the other hand, about one-third of the harvest from other food crops is sold on average and about 11 % of the area is cultivated with non-food cash crops. These numbers reveal that – in spite of their subsistence orientation – farm households in Malawi participate in market transactions and depend on agricultural cash incomes to buy goods and services that they do not produce themselves. Farm-gate sales, village markets and district markets all play important roles for smallholder crop marketing (see Table S2 in the online supplementary material).

In terms of agricultural technologies, improved maize and legume varieties are used by 81 and 62 % of the farm households, respectively. Over 90 % of the households use chemical fertilizers for crop production. Maize–legume intercropping is practised by about half of the farm households. Hence, it seems that modern inputs and improved agricultural practices have been adopted relatively widely by smallholder farmers in Malawi. This may be the result of special support and dissemination programmes run by governmental and non-governmental organizations during the last 10 years.

### Association between farm production diversity and dietary diversity

We now look at results from the regression models explained in equation (1) with dietary diversity as dependent and farm production diversity as independent variables. In Table 2, the production diversity score is used as indicator of production diversity. The production diversity score is positively associated with dietary diversity. This should not surprise in subsistence-oriented households, where a large part of what is produced on the farm is consumed in the farm household. Yet the marginal effects are relatively small. Increasing farm production diversity by one food group is associated with only a 0·12 increase in the number of food groups consumed by the farm household. For children, the marginal effect is somewhat larger (0·17), for mothers it is somewhat smaller (0·11).

**Table 2:** Association between production diversity score and dietary diversity in smallholder farm households, rural central and southern Malawi, 2014

	Household DDS	Child DDS	Mother DDS
Production diversity score	0·124***	0·168***	0·114**
se	0·048	0·056	0·048
No. of observations	408	519	408
*χ* ^2^	6·787***	9·084***	5·565**
*α* estimates of equidispersion test	−0·0921***	−0·0183	−0·0807***
se	0·0101	0·0167	0·0117

DDS, dietary diversity score. Marginal effects are shown with their village cluster-corrected standard errors. Based on equidispersion test results, the models for household and mother DDS were estimated with a generalized Poisson estimator; the model for child DDS was estimated with a standard Poisson estimator. **P<0·05, ***P<0·01.

In Table 3, results from the same type of regression models are shown, but now using the crop species count instead of the production diversity score as independent variable. The estimated marginal effects are also positive, but smaller than those in Table 2. For the household-level model, the effect is not statistically significant. Comparison between Tables 2 and 3 suggests that the number of crop species grown has a lesser influence on dietary diversity than the number of food groups produced. But regardless of the indicator used, substantial improvement in dietary diversity would require very high levels of farm production diversity if this were the only strategy pursued.

**Table 3:** Association between crop species count and dietary diversity in smallholder farm households, rural central and southern Malawi, 2014

	Household DDS	Child DDS	Mother DDS
Crop species count	0·042	0·073**	0·050*
se	0·027	0·030	0·028
No. of observations	408	519	408
*χ* ^2^	2·332	6·022**	3·306*
*α* estimates of equidispersion test	−0·0904***	−0·0161	−0·0799***
se	0·0103	0·0166	0·0118

DDS, dietary diversity score. Marginal effects are shown with their village cluster-corrected standard errors. Based on equidispersion test results, the models for household and mother DDS were estimated with a generalized Poisson estimator; the model for child DDS was estimated with a standard Poisson estimator. *P<0·1, **P<0·05, ***P<0·01.

### The role of markets

We now analyse the role of markets for dietary diversity by estimating the regression models explained in equation (2). In one set of models, we use the market access variables as covariates. In another set of models, we use the market participation variables instead. Due to the correlation between market access and market participation, including both types of variables in the same models would lead to problems of collinearity. In addition to the market variables, we include a vector of other socio-economic and demographic covariates. Results are shown in Table 4.

**Table 4:** Associations between farm production diversity, market access and dietary diversity in smallholder farm households, rural central and southern Malawi, 2014

	Market access models			Market participation models		
	Household DDS	Child DDS	Mother DDS	Household DDS	Child DDS	Mother DDS
Production diversity score	0·145***	0·189***	0·133***	0·101**	0·145**	0·087*
se	0·047	0·057	0·047	0·049	0·063	0·050
Village market	0·263	0·270	0·129			
se	0·162	0·197	0·164			
Time to district market	−0·205**	−0·194**	−0·251***			
se	0·093	0·093	0·078			
Share of maize sold				0·014**	0·015**	0·013**
se				0·006	0·006	0·006
Share of other food crops sold				0·005**	0·003	0·005**
se				0·002	0·003	0·002
Area share of non-food cash crops				−0·002	−0·006	−0·001
se				0·004	0·006	0·004
Off-farm income	0·001***	0·001***	0·001***	0·001***	0·001***	0·002***
se	0·000	0·000	0·000	0·000	0·000	0·000
Farm size	0·043	0·018	0·042	0·000	−0·003	0·003
se	0·061	0·069	0·061	0·063	0·071	0·064
Household size	−0·118***	−0·170**	−0·143***	−0·100**	−0·154**	−0·128**
se	0·045	0·069	0·050	0·047	0·066	0·051
Age of head	0·013	0·000	0·008	0·019**	0·006	0·015
se	0·009	0·011	0·009	0·009	0·011	0·009
Male head	0·017	0·065	−0·011	0·040	0·123	0·043
se	0·258	0·304	0·269	0·255	0·299	0·265
Education of head	0·061**	0·028	0·051**	0·060**	0·027	0·051**
se	0·026	0·029	0·024	0·026	0·030	0·024
No. of observations	408	519	408	408	519	408
*χ* ^2^	63·35***	44·72***	69·80***	58·74***	42·77***	67·54***
*α* estimates of equidispersion test	−0·1029***	−0·0332**	−0·0930***	−0·1023***	−0·0336**	−0·0927***
se	0·0095	0·0158	0·0109	0·0096	0·0158	0·0110

DDS, dietary diversity score Marginal effects are shown with their village cluster-corrected standard errors. Based on equidispersion test results, all models were estimated with a generalized Poisson estimator. **P<0·05, ***P<0·01.

While the village market dummy is positively associated with dietary diversity, the marginal effects are not statistically significant. Distance to the district market, however, is statistically significant in all three models (household, child and mother). The negative marginal effects imply that longer walking times to the district market are associated with lower dietary diversity. At the same time, the effects of farm production diversity on dietary diversity remain robust. Comparing the estimates suggests that reducing the walking time to the district market by 1 h would have a larger positive effect on dietary diversity than producing one additional food group on the farm. While the exact magnitude of the point estimates should not be over-interpreted, these results clearly confirm that market access matters for the dietary quality of farm households and individual household members.

**Fig. 1:**
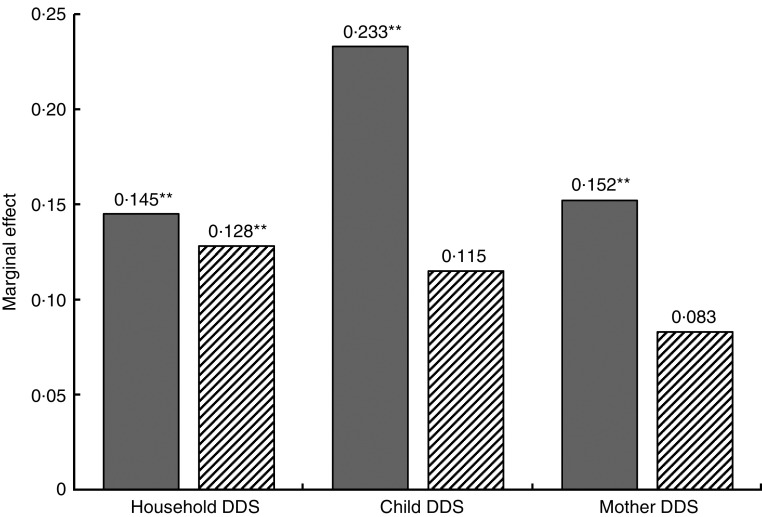
Marginal effect of production diversity score on household (*n* 408) and individual (children below 5 years, *n* 519; mothers, *n* 408) dietary diversity scores (DDS) by distance to district market (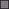, far from district market; 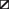, close to district market; based on mean value of the variable ‘time to district market’); smallholder farm households, rural central and southern Malawi, 2014. **P<0·05

Another interesting question is whether the effect of farm production diversity on dietary diversity differs by market access. A plausible hypothesis would be that production diversity matters more in remote settings and loses importance with better market access. To test this hypothesis, we split the sample at the mean value of the variable ‘time to district market’ and re-estimated the models for the two sub-samples. The results, which are summarized in Fig. 1, confirm this hypothesis. For households closer to the district market, the effect of farm production diversity is smaller and even turns statistically insignificant in the individual-level models.

The role of actual market participation is analysed in the models shown on the right-hand side of Table 4. The share of maize and other food crops sold is positively associated with household and individual dietary diversity. The marginal effects imply that a 10 percentage point increase in the share of maize sold is associated with a 0·14 higher household DDS, almost identical in magnitude across the three models. It seems that the cash incomes generated from maize sales are used to buy more food diversity in the market. The effects for the sale of other food crops are smaller, but also positive and significant in the models for households and mothers.

Table 4 also shows estimates for the role of other socio-economic and demographic factors. Household size is negatively associated with dietary diversity in all models. Education of the household head plays a positive role for household and mothers’ dietary diversity. Interesting to observe are also the effects of off-farm income, which are positive and highly significant in all models. This is another signal of the important role of markets for purchasing food diversity.

To test the robustness of the results, we re-estimated all models in Table 4 using the crop species count instead of production diversity scores as explanatory variable (see Table S3 in the online supplementary material). The effects of production diversity are smaller when the crop species count is used as the indicator. The other estimates in these alternative model specifications largely confirm the findings already discussed.

### The role of agricultural technologies

A final set of regression models examines the role of agricultural technology for dietary diversity, as described in equation (3). As explained, we look at four concrete technologies that are included into the models as dummy variables. Results are shown in Table 5. The estimated coefficients for the four technologies are predominantly positive, but many of these coefficients are not statistically significant. One exception is the use of chemical fertilizer, with positive and significant coefficients in the household models and the individual models for mothers. Using chemical fertilizer in crop production is associated with a 0·57 higher household DDS. This effect is larger than that of any other single factor included in the models and points at the important role of crop productivity for farm household diets. At the same time, the fertilizer effect further stresses the important role of markets. Access to input and output markets facilitates farmers’ adoption of fertilizers and other productivity-enhancing inputs.

**Table 5:** Associations between farm production diversity, market access, agricultural technology and dietary diversity in smallholder farm households, rural central and southern Malawi, 2014

	Market access models			Market participation models		
	Household DDS	Child DDS	Mother DDS	Household DDS	Child DDS	Mother DDS
Production diversity score	0·129***	0·171***	0·114**	0·087*	0·132**	0·072
se	0·047	0·058	0·048	0·049	0·063	0·050
Village market	0·231	0·213	0·088			
se	0·160	0·203	0·164			
Time to district market	−0·217**	−0·207**	−0·265***			
se	0·092	0·093	0·077			
Share of maize sold				0·013**	0·014**	0·012**
se				0·006	0·006	0·006
Share of other food crops sold				0·005**	0·003	0·005**
se				0·002	0·003	0·002
Area share of non-food cash crops				−0·002	−0·004	−0·001
se				0·004	0·007	0·004
Improved maize varieties	0·267	0·215	0·263	0·168	0·124	0·180
se	0·174	0·229	0·200	0·172	0·235	0·196
Improved legume varieties	0·098	0·087	0·113	0·001	0·004	0·009
se	0·180	0·218	0·179	0·175	0·218	0·178
Chemical fertilizer	0·567*	0·272	0·660*	0·595*	0·313	0·647*
se	0·320	0·415	0·352	0·304	0·416	0·348
Maize–legume intercropping	0·044	0·258	0·058	0·073	0·268	0·080
se	0·152	0·210	0·166	0·149	0·213	0·163
Off-farm income	0·001***	0·001***	0·001***	0·001***	0·001***	0·001***
se	0·000	0·000	0·000	0·000	0·000	0·000
Farm size	0·032	0·017	0·029	−0·004	−0·001	−0·002
se	0·063	0·072	0·063	0·064	0·073	0·065
Household size	−0·114***	−0·172**	−0·139***	−0·098**	−0·156**	−0·124**
se	0·044	0·067	0·048	0·045	0·065	0·050
Age of head	0·011	−0·000	0·006	0·018**	0·005	0·013
se	0·009	0·011	0·009	0·009	0·012	0·009
Male head	0·031	0·076	0·002	0·048	0·126	0·055
se	0·258	0·309	0·269	0·253	0·299	0·262
Education of head	0·054**	0·024	0·045*	0·053**	0·024	0·045*
se	0·026	0·030	0·024	0·026	0·030	0·024
No. of observations	408	519	408	408	519	408
*χ* ^2^	76·74***	53·05***	83·40***	66·73***	49·52***	74·57***
*α* estimates of equidispersion test	−0·1049***	−0·0355**	−0·0955***	−0·1039***	−0·0356**	−0·0948***
se	0·0094	0·0157	0·0106	0·0094	0·0157	0·0107

DDS, dietary diversity score. Marginal effects are shown with their village cluster-corrected standard errors. Based on equidispersion test results, all models were estimated with a generalized Poisson estimator. *P<0·1, **P<0·05, ***P<0·01.

Fertilizer adoption is positively correlated with the adoption of improved seeds. This correlation and the resulting inflation of the standard errors may explain why the effects for improved maize varieties are not statistically significant, in spite of the relatively large point estimates.

Again as a robustness check, we re-estimated all models in Table 5 using the crop species count instead of production diversity scores as explanatory variable (see Table S4 in the online supplementary material). Except for the smaller effects of the crop species count, the other estimates are similar. Furthermore, as the adoption of agricultural technologies may influence farm production diversity and vice versa, we also re-estimated the same models but excluding farm production diversity (see Table S5 in the online supplementary material). The estimates for the remaining variables do not change much, meaning that the main results are fairly robust to changes in model specification.

## Discussion

We have analysed the role of farm production diversity, market access and technology adoption for dietary diversity in smallholder farm households in Malawi. Even though we used different data and indicators of dietary diversity, our results are in line with those from previous studies^(^ ^19^ ^,^ ^27^ ^–^ ^29^ ^)^. Yet the analysis also offers a few new insights.

Our results confirm that production diversity is positively associated with dietary diversity. But the effect is relatively small. Previous studies measured production diversity in terms of a simple species count, which we also did in some of the model specifications. However, in the main specifications we used production diversity scores, defined as the number of food groups produced. When using production diversity scores instead of a species count, the effect on dietary diversity is larger. This is plausible in a subsistence-oriented setting like rural Malawi, where a significant share of what is produced on the farm is consumed in the farm household. Interestingly, the opposite was found in a previous study that had used data from more commercially oriented farms in Indonesia, Kenya and Uganda^(^ ^27^ ^)^. In more commercialized settings with better market access, increasing the number of food groups produced on a farm may entail lower cash revenues and foregone benefits from specialization^(^ ^32^ ^)^.

But even in a more subsistence-oriented setting like rural Malawi we found an important role of markets for dietary diversity. Closer proximity to markets does not only contribute to higher dietary diversity, but also tends to reduce the effect of farm production diversity. Farm households use markets to sell agricultural produce and to buy foods that they do not or cannot produce themselves. Even foods that are produced on the farm may not always be stored for the entire year; issues of seasonality are particularly important for fresh fruits and vegetables^(^ ^21^ ^)^. Previous studies have shown that foods purchased from the market contribute considerably to farm household diets also in subsistence-oriented settings^(^ ^19^ ^,^ ^20^ ^)^. We also found that sales of maize and other crops have positive effects, because the cash income thus generated can be used to purchase diverse foods in the market. Finally, the analysis has shown that the adoption of modern agricultural technologies is positively associated with dietary diversity. These results clearly suggest that commercial orientation and productivity-enhancing innovation are conducive for better nutrition in smallholder farm households.

Most previous studies that had analysed the role of farm production diversity and other factors for dietary diversity used food consumption data collected at the household level. In the present study, we have used 24 h recall data collected at household and individual levels for young children and mothers. The estimation results for the household- and individual-level models were surprisingly similar. Hence, results do not seem to be driven by the method of measurement of dietary intakes. This is good news for researchers wishing to use secondary data sources. Many nationally representative living standard measurement survey nowadays contain food consumption recalls at the household level, whereas individual-level recall data are available only from more specialized surveys that have a particular nutrition focus.

However, it should be stressed that cross-sectional data collected in a single round have limitations when they are used to assess diets. This applies to secondary data as well as to the primary data collected and used in the present study. In particular, single-round data do not reflect seasonal variation in dietary patterns that can be very important in rural areas. Our data were collected during the lean season prior to the main agricultural harvest. This means that the role of own farm production for dietary diversity may be smaller than during harvest or post-harvest periods. To capture seasonality effects, repeated surveys carried out during different times of the year would be required.

## Conclusion

Farm production diversity is positively associated with dietary diversity in Malawi. Hence, on-farm crop diversification may help to improve household diets to some extent. However, the magnitude of the estimates suggests that the positive dietary effects of further diversifying on-farm production will be relatively small. Access to markets for buying food and for selling farm produce, as well as the adoption of modern agricultural technology, were shown to be more important for dietary quality. Hence, improving access to markets through better infrastructure and institutions and promoting the spread of productivity-enhancing technologies seem to be more promising approaches to improve farm household diets. If diversification is pursued, it should not obstruct smallholder market integration and commercialization.

Different models were used, comparing effects on DDS at household and individual levels. Overall, the results were similar across the different models. This similarity suggests that household-level food consumption data, which are more often available from secondary statistics than individual-level data, can be used for broader questions of dietary quality without introducing a significant bias. Of course, for planning interventions that focus on particular target groups, more detailed individual-level data will be required.

## Supplementary material

For supplementary material accompanying this paper visit https://doi.org/10.1017/S1368980016002135. click here to view supplementary material

## References

[1] Food and Agriculture Organization of the United Nations (2015) The State of Food Insecurity in the World. Rome: FAO.

[2] International Food Policy Research Institute (2016) Global Nutrition Report 2016. From Promise to Impact: Ending Malnutrition by 2030. Washington, DC: IFPRI.

[3] Black RE, Victora CG, Walker SP et al. (2013) Maternal and child undernutrition and overweight in low-income and middle-income countries. Lancet 382, 427–451. 23746772 10.1016/S0140-6736(13)60937-X

[4] Frelat R, Lopez-Ridaura S, Giller KE et al. (2016) Drivers of household food availability in sub-Saharan Africa based on big data from small farms. Proc Natl Acad Sci USA 113, 458–463. 26712016 10.1073/pnas.1518384112 PMC4720294

[5] Pinstrup-Andersen P (2007) Agricultural research and policy for better health and nutrition in developing countries: a food systems approach. Agric Econ 37, 187–198.

[6] Pingali P (2015) Agricultural policy and nutrition outcomes – getting beyond the preoccupation with staple grains. Food Sec 7, 583–591.

[7] Khoury CK, Bjorkman AD, Dempewolf H et al. (2014) Increasing homogeneity in global food supplies and the implications for food security. Proc Natl Acad Sci USA 111, 4001–4006. 24591623 10.1073/pnas.1313490111 PMC3964121

[8] Graham RD, Welch RM, Saunders DA et al. (2007) Nutritious subsistence food systems. In Advances in Agronomy, vol. 92, pp. 1–72 [DL Sparks, editor]. New York: Academic Press.

[9] Frison EA, Smith IF, Johns T et al. (2006) Agricultural biodiversity, nutrition, and health: making a difference to hunger and nutrition in the developing world. Food Nutr Bull 27, 167–179. 16786983 10.1177/156482650602700208

[10] M’Kaibi FK, Steyn NP, Ochola S et al. (2015) Effects of agricultural biodiversity and seasonal rain on dietary adequacy and household food security in rural areas of Kenya. BMC Public Health 15, 422. 25909468 10.1186/s12889-015-1755-9 PMC4437678

[11] Bezner Kerr R, Berti PR & Shumba L (2011) Effects of a participatory agriculture and nutrition education project on child growth in northern Malawi. Public Health Nutr 14, 1466–1472. 21059284 10.1017/S1368980010002545

[12] Savy M, Martin-Prével Y, Traissac P et al. (2006) Dietary diversity scores and nutritional status of women change during the seasonal food shortage in rural Burkina Faso. J Nutr 136, 2625–2632. 16988137 10.1093/jn/136.10.2625

[13] Steyn NP, Nel JH, Nantel G et al. (2006) Food variety and dietary diversity scores in children: are they good indicators of dietary adequacy? Public Health Nutr 9, 644–650. 16923296 10.1079/phn2005912

[14] Berry EM, Dernini S, Burlingame B et al. (2015) Food security and sustainability: can one exist without the other? Public Health Nutr 18, 2293–2302. 25684016 10.1017/S136898001500021X PMC10271846

[15] Herforth A (2015) Access to adequate nutritious food: new indicators to track progress and inform action. In The Fight Against Hunger and Malnutrition: The Role of Food, Agriculture, and Targeted Policies, pp. 139–162 [D Sahn, editor]. Oxford: Oxford University Press.

[16] Bowman MS & Zilberman D (2013) Economic factors affecting diversified farming systems. Ecol Soc 18, 33.

[17] Webb P & Kennedy E (2014) Impacts of agriculture on nutrition: nature of the evidence and research gaps. Food Nutr Bull 35, 126–132. 24791585 10.1177/156482651403500113

[18] Food and Agriculture Organization of the United Nations (2014) The State of Food and Agriculture: Innovation in Family Farming. Rome: FAO.

[19] Sibhatu KT, Krishna VV & Qaim M (2015) Production diversity and dietary diversity in smallholder farm households. Proc Natl Acad Sci USA 112, 10657–10662. 26261342 10.1073/pnas.1510982112 PMC4553771

[20] Luckett B, DeClerck F, Fanzo J et al. (2015) Application of the nutrition functional diversity indicator to assess food system contributions to dietary diversity and sustainable diets of Malawian households. Public Health Nutr 18, 2479–2487. 26027595 10.1017/S136898001500169X PMC10271332

[21] Hirvonen K, Taffesse AS & Hassen IW (2016) Seasonality and household diets in Ethiopia. Public Health Nutr 19, 1723–1730. 26585676 10.1017/S1368980015003237 PMC10271090

[22] Hirvonen K & Hoddinott J (2014) Agricultural Production and Children’s Diets: Evidence from Rural Ethiopia. Ethiopia Strategy Support Program Working Paper no. 69. Washington, DC: International Food Policy Research Institute.

[23] Barrett CB (2008) Smallholder market participation: concepts and evidence from eastern and southern Africa. Food Policy 33, 299–317.

[24] Johns T, Powell B, Maundu P et al. (2013) Agricultural biodiversity as a link between traditional food systems and contemporary development, social integrity and ecological health. J Sci Food Agric 93, 3433–3442. 23963831 10.1002/jsfa.6351

[25] Powell B, Maundu P, Kuhnlein HV et al. (2013) Wild foods from farm and forest in the East Usambara mountains, Tanzania. Ecol Food Nutr 52, 451–478. 24083514 10.1080/03670244.2013.768122

[26] Bharucha Z & Pretty JN (2010) The roles and values of wild foods in agricultural systems. Philos Trans R Soc Lond B Biol Sci 365, 2913–2926. 20713393 10.1098/rstb.2010.0123 PMC2935111

[27] Sibhatu KT & Qaim M (2016) Farm Production Diversity and Dietary Quality: Linkages and Measurement Issues. GlobalFood Discussion Paper no. 80. Goettingen: University of Goettingen.

[28] Snapp SS & Fisher M (2015) ‘Filling the maize basket’ supports crop diversity and quality of household diet in Malawi. Food Sec 7, 83–96.

[29] Jones AD, Shrinivas A & Bezner Kerr R (2014) Farm production diversity is associated with greater household dietary diversity in Malawi: findings from nationally representative data. Food Policy 46, 1–12.

[30] Berti PR (2015) Relationship between production diversity and dietary diversity depends on how number of foods is counted. Proc Natl Acad Sci USA 112, E5656. 26432884 10.1073/pnas.1517006112 PMC4620869

[31] Remans R, DeClerck F, Kennedy G et al. (2015) Expanding the view on the production and consumption diversity link: scale, function and change over time. Proc Natl Acad Sci USA 112, e6082. 26515099 10.1073/pnas.1518531112 PMC4653189

[32] Sibhatu KT, Krishna VV & Qaim M (2015) Reply to Berti: relationship between production and consumption diversity remains small also with modified diversity measures. Proc Natl Acad Sci USA 112, e5657. 26432883 10.1073/pnas.1517209112 PMC4620863

[33] de Haen H, Klasen S & Qaim M (2011) What do we really know? Metrics for food insecurity and undernutrition. Food Policy 36, 760–769.

[34] Ecker O & Qaim M (2011) Analyzing nutritional impacts of policies: an empirical study for Malawi. World Dev 39, 412–428.

[35] Headey D & Ecker O (2013) Rethinking the measurement of food security: from first principles to best practice. Food Sec 5, 327–343.

[36] Kennedy G, Berardo A, Papavero C et al. (2010) Proxy measures of household food consumption for food security assessment and surveillance: comparison of the household dietary diversity and food consumption scores. Public Health Nutr 13, 2010–2018. 20602864 10.1017/S136898001000145X

[37] Ruel MT (2003) Operationalizing dietary diversity: a review of measurement issues and research priorities. J Nutr 133, 11 Suppl. 2, 3911S–3926S. 14672290 10.1093/jn/133.11.3911S

[38] Herforth A (2010) Promotion of traditional African vegetables in Kenya and Tanzania: a case study of an intervention representing emerging imperatives in global nutrition. Doctoral Dissertation, Cornell University.

[39] Olney KD, Talukder A, Iannotti LL et al. (2009) Assessing impact and impact pathways of a homestead food production program on household and child nutrition in Cambodia. Food Nutr Bull 30, 355–369. 20496626 10.1177/156482650903000407

[40] Gibson RS (2005) Principles of Nutritional Assessment. New York: Oxford University Press.

[41] Ellis F & Manda E (2012) Seasonal food crisis and policy responses: a narrative account of three food security crises in Malawi. World Dev 40, 1407–1417.

[42] Johnson KB, Jacob A & Brown ME (2013) Forest cover associated with improved child health and nutrition: evidence from the Malawi Demographic and Health Survey and satellite data. Glob Health Sci Pract 1, 237–248. 25276536 10.9745/GHSP-D-13-00055 PMC4168570

[43] Haug A, Christophersen OA, Kinabo J et al. (2010) Use of dried kapenta (*Limnothrissa miodon* and *Stolsothrissa tanganicae*) and other products based on whole fish for complementing maize-based diets. Afr J Food Agric Nutr Dev 10, issue 5; available at http://www.ajol.info/index.php/ajfand/issue/view/7424

[44] Akinnifesi FK, Kwesiga FR, Mhango J et al. (2004) Domesticating priority Miombo indigenous fruit trees as promising livelihood option for smallholder farmers in Southern Africa. Acta Hort 632, 15–30.

[45] Ruel MT, Harris J & Cunningham K (2012) Measuring dietary quality in developing countries: a review of the usefulness of individual dietary diversity indicators. In Diet Quality: An Evidence-Based Approach, pp. 239–261 [VR Preedy, editor]. New York: Springer.

[46] Kennedy G, Ballard T & Dop MC (2011) Guidelines for Measuring Household and Individual Dietary Diversity. Rome: FAO.

[47] Keding GB, Msuya JM, Maass BL et al. (2012) Relating dietary diversity and food variety scores to vegetable production and socio-economic status of women in rural Tanzania. Food Sec 4, 129–140.

[48] Swindale A & Bilinsky P (2006) Household Dietary Diversity Score (HDDS) for Measurement of Household Food Access: Indicator Guide (V.2). Washington, DC: FHI 360/Food and Nutrition Technical Assistance III Project.

[49] Malapit HJL, Kadiyala S, Quisumbing AR et al. (2015) Women’s empowerment mitigates the negative effects of low production diversity on maternal and child nutrition in Nepal. J Dev Stud 51, 1097–1123.

[50] Shiferaw B, Kassie M, Jaleta M et al. (2014) Adoption of improved wheat varieties and impacts on household food security in Ethiopia. Food Policy 44, 272–284.

[51] Qaim M & Kouser S (2013) Genetically modified crops and food security. PLoS ONE 8, e64879. 23755155 10.1371/journal.pone.0064879 PMC3674000

[52] Chirwa EW & Dorward A (2013) Agricultural Input Subsidies: The Recent Malawi Experience. Oxford: Oxford University Press.

[53] Mhango WG, Snapp SS & Phiri GYK (2013) Opportunities and constraints to legume diversification for sustainable maize production on smallholder farms in Malawi. Renew Agric Food Syst 28, 234–244.

[54] Bezner Kerr R, Snapp S, Chirwa M et al. (2007) Participatory research on legume diversification with Malawian smallholder farmers for improved human nutrition and soil fertility. Exp Agric 43, 437–453.

[55] Greene WH (2012) Econometric Analysis, 7th ed. Boston, MA: Prentice-Hall.

[56] Cameron AC & Trivedi PK (2013) Regression Analysis of Count Data, 2nd ed. Cambridge: Cambridge University Press.

[57] Harris T, Yang Z & Hardin JW (2012) Modeling underdispersed count data with generalized Poisson regression. Stata J 12, 736–747.

[58] Cameron AC & Miller DL (2015) A practitioner’s guide to cluster-robust inference. J Hum Res 50, 317–372.

