# Supplementary material for: Farm production, market access and dietary diversity in Malawi
Source: Public Health Nutr. 2016 Sep 9;20(2):325–35. doi: 10.1017/S1368980016002135 (PMC5244442; doi:10.1017/S1368980016002135)
Supplement: Supplementary file 1 [file S1368980016002135sup001.docx]

**Online Supplementary Material**

Table S1: Percentage share of food groups consumed among farm households and individuals

| Food group | Households | Children | Mothers |
| --- | --- | --- | --- |
| Cereals | 99 | 90 | 98 |
| Vegetables | 82 | 73 | 80 |
| Miscellaneous^†^ | 62 | 58 | 61 |
| Fruits | 51 | 51 | 51 |
| Oils and fats | 31 | 29 | 31 |
| Sugar and honey | 30 | 28 | 30 |
| Legumes and nuts | 22 | 21 | 21 |
| Fish | 20 | 19 | 20 |
| Tubers and roots | 7 | 6 | 7 |
| Meat and poultry | 6 | 7 | 6 |
| Eggs | 4 | 3 | 4 |
| Milk and milk products | 3 | 3 | 3 |

† Miscellaneous includes spices, condiments and beverages.

Table S2: Importance of different marketing channels for crop sales^†^

| Marketing channel | Total number of sales during last season | Share of sales in percent |
| --- | --- | --- |
| Farm gate sales | 233 | 31 |
| Village market sales | 200 | 26 |
| District market sales | 323 | 43 |

† 84 percent (341 of 408) of the sample farms sold crops during the last season prior to the survey.

Table S3: Crop species count, market access and dietary diversity

|  | Market access models | | | Market participation models | | |
| --- | --- | --- | --- | --- | --- | --- |
|  | Household DDS | Child DDS | Mother DDS | Household DDS | Child DDS | Mother DDS |
| Crop species count | 0.058*  (0.030) | 0.095***  (0.034) | 0.063**  (0.030) | 0.035  (0.027) | 0.075**  (0.034) | 0.044  (0.028) |
| Village market | 0.326* | 0.364* | 0.207 |  |  |  |
|  | (0.170) | (0.210) | (0.169) |  |  |  |
| Time to district market | -0.202**  (0.093) | -0.193**  (0.095) | -0.248***  (0.079) |  |  |  |
| Share of maize sold |  |  |  | 0.015**  (0.006) | 0.016***  (0.006) | 0.014**  (0.006) |
| Share of other food crops sold |  |  |  | 0.005**  (0.002) | 0.003  (0.003) | 0.006**  (0.002) |
| Area share of non-food cash crops |  |  |  | -0.002  (0.004) | -0.006  (0.006) | -0.002  (0.004) |
| Livestock | 0.063 | 0.122** | 0.087* | 0.034 | 0.100* | 0.058 |
|  | (0.046) | (0.053) | (0.045) | (0.050) | (0.057) | (0.051) |
| Off-farm income | 0.001*** | 0.001*** | 0.002*** | 0.001*** | 0.001*** | 0.002*** |
|  | (0.000) | (0.000) | (0.000) | (0.000) | (0.000) | (0.000) |
| Farm size | 0.040 | -0.006 | 0.031 | -0.001 | -0.027 | -0.009 |
|  | (0.062) | (0.069) | (0.061) | (0.063) | (0.068) | (0.063) |
| Household size | -0.134*** | -0.196*** | -0.165*** | -0.109** | -0.172*** | -0.142*** |
|  | (0.044) | (0.067) | (0.049) | (0.046) | (0.064) | (0.050) |
| Age of head | 0.012 | -0.001 | 0.008 | 0.019** | 0.005 | 0.015* |
|  | (0.008) | (0.011) | (0.009) | (0.009) | (0.011) | (0.009) |
| Male head | 0.036 | 0.080 | -0.001 | 0.052 | 0.122 | 0.043 |
|  | (0.254) | (0.300) | (0.267) | (0.253) | (0.294) | (0.264) |
| Education of head | 0.054**  (0.026) | 0.017  (0.029) | 0.044*  (0.023) | 0.056**  (0.026) | 0.019  (0.030) | 0.046*  (0.024) |
|  |  |  |  |  |  |  |
| Observations | 408 | 519 | 408 | 408 | 519 | 408 |
| Chi2 | 73.12*** | 51.23*** | 76.94*** | 58.74*** | 47.11*** | 67.43*** |
| α estimates of equi-dispersion test | -0.1016***  (0.0095) | -0.0331**  (0.0153) | -0.0931***  (0.0107) | -0.1017***  (0.0096) | -0.0339**  (0.0155) | -0.0929***  (0.0109) |

DDS, dietary diversity score.

Marginal effects are shown with village cluster-corrected SEs in parentheses. Based on equi-dispersion test results, all models were estimated with a generalized Poisson estimator.

***P<0.01, **P<0.05, *P<0.1

Table S4: Crop species count, market access, agricultural technology and dietary diversity

|  | Market access models | | | Market participation models | | |
| --- | --- | --- | --- | --- | --- | --- |
|  | Household DDS | Child DDS | Mother DDS | Household DDS | Child DDS | Mother DDS |
| Crop species count | 0.047  (0.029) | 0.083**  (0.034) | 0.051*  (0.030) | 0.031  (0.028) | 0.071**  (0.034) | 0.040  (0.028) |
| Village market | 0.279* | 0.295 | 0.152 |  |  |  |
|  | (0.166) | (0.215) | (0.167) |  |  |  |
| Time to district market | -0.216**  (0.092) | -0.208**  (0.094) | -0.264***  (0.078) |  |  |  |
| Share of maize sold |  |  |  | 0.013**  (0.006) | 0.015**  (0.006) | 0.012**  (0.006) |
| Share of other food crops sold |  |  |  | 0.005**  (0.002) | 0.003  (0.003) | 0.006**  (0.002) |
| Area share of non-food cash crops |  |  |  | -0.003  (0.004) | -0.005  (0.006) | -0.002  (0.004) |
| Improved maize varieties | 0.254  (0.175) | 0.245  (0.225) | 0.263  (0.200) | 0.153  (0.173) | 0.141  (0.231) | 0.173  (0.194) |
| Improved legume varieties | 0.098  (0.175) | 0.071  (0.214) | 0.102  (0.175) | -0.002  (0.172) | -0.024  (0.215) | -0.008  (0.176) |
| Chemical fertilizer | 0.634**  (0.316) | 0.340  (0.399) | 0.706**  (0.344) | 0.656**  (0.300) | 0.380  (0.399) | 0.688**  (0.339) |
| Maize-legume intercropping | 0.065  (0.153) | 0.290  (0.205) | 0.073  (0.165) | 0.087  (0.149) | 0.299  (0.208) | 0.087  (0.162) |
| Livestock | 0.067 | 0.131** | 0.091** | 0.035 | 0.104* | 0.058 |
|  | (0.046) | (0.054) | (0.045) | (0.050) | (0.056) | (0.051) |
| Off-farm income | 0.001*** | 0.001*** | 0.001*** | 0.001*** | 0.001*** | 0.001*** |
|  | (0.000) | (0.000) | (0.000) | (0.000) | (0.000) | (0.000) |
| Farm size | 0.028 | -0.009 | 0.017 | -0.006 | -0.027 | -0.015 |
|  | (0.064) | (0.071) | (0.063) | (0.064) | (0.070) | (0.064) |
| Household size | -0.130*** | -0.199*** | -0.159*** | -0.106** | -0.175*** | -0.138*** |
|  | (0.043) | (0.066) | (0.047) | (0.045) | (0.063) | (0.049) |
| Age of head | 0.010 | -0.001 | 0.006 | 0.017** | 0.005 | 0.013 |
|  | (0.008) | (0.011) | (0.009) | (0.009) | (0.011) | (0.009) |
| Male head | 0.044 | 0.086 | 0.007 | 0.057 | 0.121 | 0.050 |
|  | (0.255) | (0.306) | (0.267) | (0.251) | (0.294) | (0.259) |
| Education of head | 0.048*  (0.026) | 0.013  (0.029) | 0.038  (0.023) | 0.050*  (0.026) | 0.015  (0.030) | 0.040*  (0.024) |
|  |  |  |  |  |  |  |
| Observations | 408 | 519 | 408 | 408 | 519 | 408 |
| Chi2 | 85.77*** | 56.89*** | 86.77*** | 68.67*** | 56.79*** | 76.36*** |
| α estimates of equi-dispersion test | -0.1038***  (0.0094) | -0.0361**  (0.0153) | -0.0957***  (0.0105) | -0.1035***  (0.0095) | -0.0364**  (0.0154) | -0.0951***  (0.0106) |

DDS, dietary diversity score.

Marginal effects are shown with village cluster-corrected SEs in parentheses. Based on equi-dispersion test results, all models were estimated with a generalized Poisson estimator.

***P<0.01, **P<0.05, *P<0.1

Table S5: Market access, agricultural technology and dietary diversity

|  | Market access models | | | Market participation models | | |
| --- | --- | --- | --- | --- | --- | --- |
|  | Household DDS | Child DDS | Mother DDS | Household DDS | Child DDS | Mother DDS |
| Village market | 0.197 | 0.149 | 0.057 |  |  |  |
|  | (0.163) | (0.210) | (0.164) |  |  |  |
| Time to district market | -0.206**  (0.091) | -0.198**  (0.098) | -0.252***  (0.078) |  |  |  |
| Share of maize sold |  |  |  | 0.013**  (0.006) | 0.016***  (0.006) | 0.013**  (0.006) |
| Share of other food crops sold |  |  |  | 0.006**  (0.002) | 0.004  (0.003) | 0.006***  (0.002) |
| Area share of non-food cash crops |  |  |  | -0.002  (0.004) | -0.004  (0.007) | -0.001  (0.004) |
| Improved maize varieties | 0.237  (0.176) | 0.183  (0.232) | 0.236  (0.202) | 0.151  (0.173) | 0.106  (0.239) | 0.165  (0.196) |
| Improved legume varieties | 0.151  (0.178) | 0.162  (0.227) | 0.160  (0.177) | 0.027  (0.174) | 0.044  (0.225) | 0.030  (0.176) |
| Chemical fertilizer | 0.682**  (0.317) | 0.436  (0.401) | 0.769**  (0.346) | 0.670**  (0.303) | 0.421  (0.406) | 0.713**  (0.342) |
| Maize-legume intercropping | 0.084  (0.157) | 0.306  (0.218) | 0.095  (0.169) | 0.095  (0.151) | 0.298  (0.219) | 0.098  (0.165) |
| Off-farm income | 0.001*** | 0.001** | 0.001*** | 0.001*** | 0.001** | 0.001*** |
|  | (0.000) | (0.000) | (0.000) | (0.000) | (0.000) | (0.000) |
| Farm size | 0.055 | 0.043 | 0.050 | 0.012 | 0.016 | 0.011 |
|  | (0.063) | (0.073) | (0.063) | (0.063) | (0.074) | (0.065) |
| Household size | -0.114*** | -0.175** | -0.139*** | -0.097** | -0.156** | -0.124** |
|  | (0.044) | (0.069) | (0.048) | (0.045) | (0.065) | (0.050) |
| Age of head | 0.009 | -0.002 | 0.005 | 0.017* | 0.004 | 0.012 |
|  | (0.008) | (0.012) | (0.009) | (0.009) | (0.012) | (0.009) |
| Male head | 0.065 | 0.120 | 0.033 | 0.073 | 0.163 | 0.075 |
|  | (0.262) | (0.319) | (0.274) | (0.254) | (0.302) | (0.263) |
| Education of head | 0.053**  (0.026) | 0.024  (0.030) | 0.044*  (0.024) | 0.053**  (0.026) | 0.023  (0.030) | 0.044*  (0.024) |
|  |  |  |  |  |  |  |
| Observations | 408 | 519 | 408 | 408 | 519 | 408 |
| Chi2 | 71.49*** | 40.31*** | 73.57*** | 66.65*** | 40.65*** | 71.29*** |
| α estimates of equi-dispersion test | -0.1024***  (0.0095) | -0.0307*  (0.0158) | -0.0937***  (0.0107) | -0.1030***  (0.0095) | -0.0332**  (0.0158) | -0.0942***  (0.0108) |

DDS, dietary diversity score.

Marginal effects are shown with village cluster-corrected SEs in parentheses. Based on equi-dispersion test results, all models were estimated with a generalized Poisson estimator.

***P<0.01, **P<0.05, *P<0.1
